# Silver diamine fluoride versus sodium fluoride for arresting dentine caries in children: a systematic review and meta-analysis

**DOI:** 10.1038/s41598-019-38569-9

**Published:** 2019-02-14

**Authors:** Alice Trieu, Ahmed Mohamed, Edward Lynch

**Affiliations:** 1 Pediatric Dental Resident, Pediatric Dentistry Department, University of Nevada, Las Vegas (UNLV), 89106 USA; 2 Visiting Faculty, Biomedical and Clinical Research, University of Nevada, Las Vegas (UNLV), 89106 USA; 3 Professor and Principal Director of Biomedical and Clinical Research, University of Nevada, Las Vegas (UNLV), 89106 USA

## Abstract

Dental caries can compromise quality of life and is associated with demineralization of tooth structure by organic acids produced by microorganisms. This study systematically reviewed the dentine caries arrest capabilities of silver diamine fluoride (SDF) and sodium fluoride (NaF). A comprehensive search strategy was developed to identify the relevant publications in electronic databases and hand searched journals and reviews (to March 2018). By applying strict inclusion and exclusion criteria, only six papers (two randomized controlled trials, two follow-up articles and two secondary statistical analysis studies) were considered for full text qualitative and quantitative assessment. The included studies were critically appraised and statistically evaluated. Only four articles were considered for meta-analysis, as the other two were secondary analyses of included studies. When comparing the caries arrest lesions of SDF and NaF, SDF was found to be statistically more effective in dentine caries arrest of primary teeth during the 18 and 30 month clinical examinations. The weighted total effect size of the differences between SDF and NaF regarding arrested caries surfaces was calculated and showed nearly double the effectiveness of SDF to NaF at 30 months. Therefore, SDF is a more effective caries management reagent than NaF. Further clinical research is needed to consolidate the findings of this systematic review.

## Introduction

Dental caries is associated with demineralization of tooth structure by organic acids produced by microorganisms, which can progress from the outer tooth structure towards the inner vital tissue causing pain and swelling^1^. In the United States, dental caries is the single most common chronic childhood disease, with more than half of children experiencing one or more decayed or filled surfaces by the age of five and this prevalence increases to more than 78% amongst 17 year olds^2^. Untreated caries can result in life threatening conditions requiring hospitalization^1^. For instance, toddlers with untreated caries exhibit poor quality of life involving pain, eating and drinking difficulties, sleeping difficulty and a sense of guilt amongst the family^3^, while pre-school aged children also experience disturbed sleep, stunted growth, higher risk of hospitalizations and emergency dental visits and increased absence from school and reduced learning ability^4^. Therefore comprehensive evidence based approaches must be developed and implemented for management of dental caries.

Currently, management of dental caries involves preventive and non-preventive treatment methods. While non-preventive caries management involves stopping or slowing the disease progress by mechanical caries removal and restoration of decayed tooth structure, preventive caries protocols, on the other hand, are implemented to prevent the onset of caries and protect the teeth from the conditions that favor the harmful impacts of oral biofilm. These protocols include: nutritional counseling, fluoride use, oral hygiene instructions, topical antimicrobial agents and the use of sugar alcohol sweeteners such as xylitol^5^. In addressing carious lesions, prevention by routine dental visits, oral hygiene and nutritional counseling resulted in a reduced number of non-preventive dental visits^6^.

Topical fluorides, such as NaF varnish, are used as preventive reagents because of their remineralization and antimicrobial abilities^7^. In 2014, the FDA approved SDF use for tooth sensitivity, with an off-label use in caries treatment and prevention and additionally approved the marketing of SDF with potassium iodide (Riva Star, SDI Limited) in 2018. SDF (38% Ag (NH_3_)_2_F) is a colourless liquid composed of 24–29% silver and 5–6% fluoride. It is also an alkaline reagent with pH 10^9^, which provides an unfavorable environment for dentine collagen enzyme activation^8^. Also, silver has been used as a medical antimicrobial since the 17^th^ century^9^ and in dentistry during 1917^10^.

Mei *et al*.^11^ demonstrated the ability of 38% SDF to inhibit demineralization and promote preservation of collagen of demineralized dentine by forming a protective layer on and within the dentinal tubules *in vitro*^11^. The latter findings suggest that SDF could be an effective preventive protocol and potentially a replacement of the commonly used NaF. This however requires a systematic and comprehensive assessment of evidence into the clinical efficacy of each reagent in managing dentine caries.

Therefore, the aim of this study was to systematically review the dentine caries arrest capabilities of SDF and NaF.

## Results

### Study Selection

**Figure 1:**
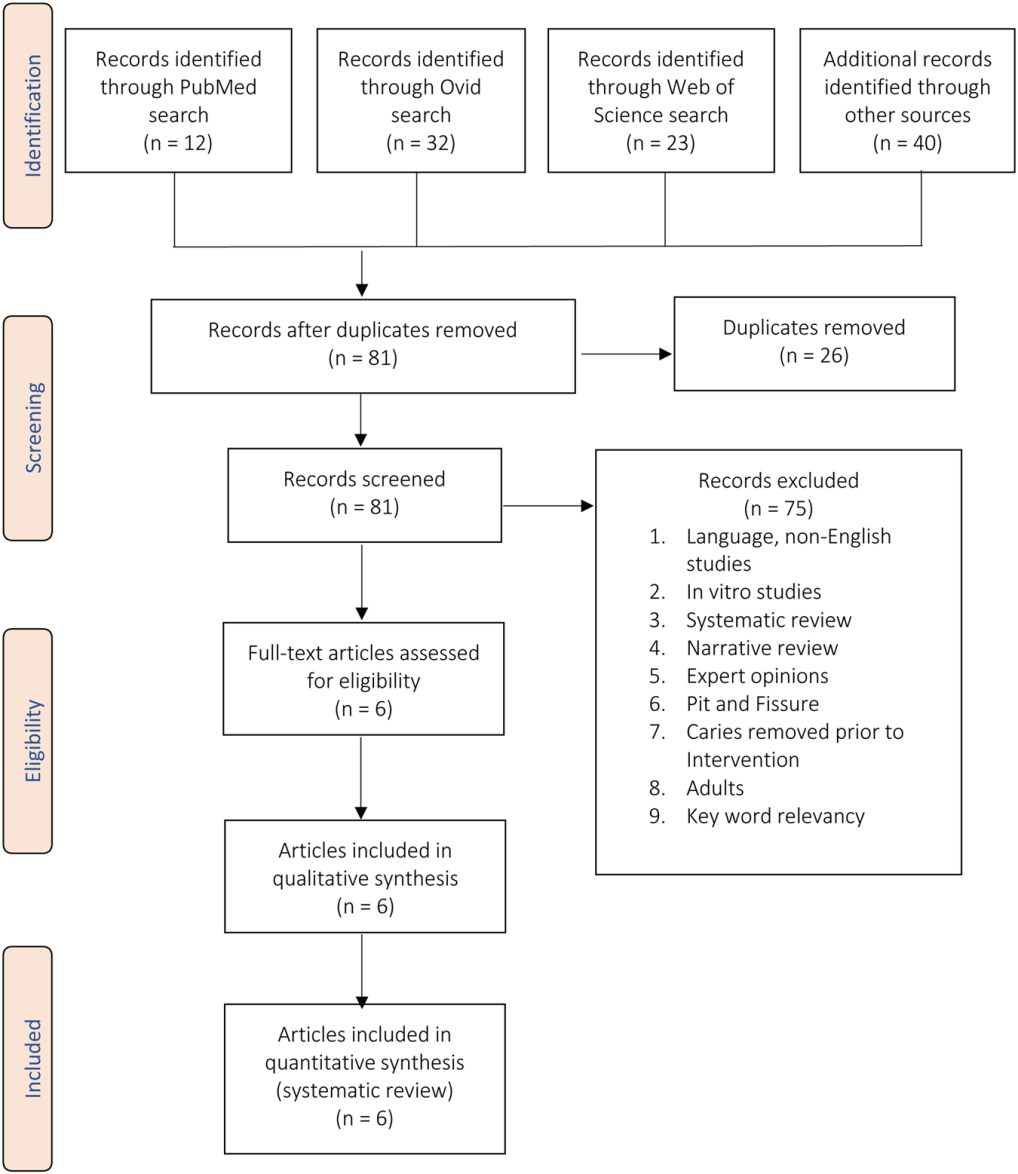
Flow diagram of study selection process.

A literature search was performed through electronic databases: Ovid, PubMed, Web of Science, and Cochrane Library and yielded 67 articles. In addition, hand searching through relevant journals and reviews resulted in 40 additional articles. The titles of the identified studies were reviewed, and 26 duplicates were excluded. Then, the abstracts and full text of the remaining 81 abstracts were independently reviewed by two investigators (Trieu and Mohamed) to determine whether an individual study was eligible for full text qualitative and quantitative analyses in this systematic review. The inclusion criteria were as follows: (1) cohort or case-control or controlled or randomized control study designs (2) included the previously mentioned key words (3) only articles published in English and (4) studies performed in humans. The exclusion criteria were as follows: (1) non-English articles (2) *in vitro* studies (3) narrative reviews and (4) expert opinions (5) enamel caries (6) adults greater than 12 years old. For detailed information on inclusion and exclusion criteria, please refer to Table 1. Disagreements among the two investigators were resolved by a third independent reviewer (Lynch). As a result, 75 articles were excluded as per the exclusion criteria and the remaining six articles were then subjected to full-text qualitative and quantitative analyses. Please refer to Fig. 1 for detailed information on study selection process.

**Table 1:** Inclusion and exclusion criteria.

Inclusion	Exclusion
*• In vivo*	*• In vitro*
• Dentin caries, Dental caries	• Non-English
• Silver diamine fluoride	• Systematic review
• Fluoride varnish	• Narrative review
• Caries activity tests	• Expert opinions
• Decayed, missing, filled (DMF)	• Pit ad fissure/non-cavitated lesions
	• Restored caries
	• Adult > 12 years old

### Study characteristics

Through the literature search, six articles met inclusion criteria. They were published between 2001 and 2018 and included four randomized controlled trials (RCT)^12–15^ and two studies which were secondary statistical analyses of RCTs^16,17^. However, the four identified RCTs consist of two individual studies (Lo *et al*.^14^ and Duangthip *et al*.^13^), while the remaining two RCTs were their follow-up articles reporting results at later time points (Chu *et al*.^15^ and Duangthip *et al*.^12^).

The included studies recruited a total of 746 pre-school children with a mean age of 3.4 years^12,13^ and 4.0 years^14,15^. The RCT by Duangthip *et al*.^13^ and its follow-up article (Duangthip *et al*.^12^) reported sample size calculations^12,13^, while the other RCT and its follow-up article did not^14,15^. Dropout rates were recorded in all articles.

Of the studies, the RCT by Lo *et al*.^14^ and its follow-up article focused on carious lesions of upper anterior primary teeth only^14,15^, while the other RCT by Duangthip *et al*.^13^ and its follow-up article included both anterior and posterior primary teeth^12,13^. Lo *et al*.^14^ and its follow-up article used 38% SDF^14,15^, while Duangthip *et al*.^13^ and its follow-up article used 30% SDF, but all RCTs compared the SDF intervention to 5% NaF varnish. All RCTs utilized the decayed, missing, and filled surfaces (DMFS) protocol to record outcomes by tactile examination. Lesions were classified as active caries or arrested caries using sharp sickled probes^14,15^ or ball ended probes^12,13^. Oral hygiene (OH) at home was evaluated by parental questionnaire in all RCTs, and clinically examined and recorded by using a visible plaque index (VPI) in one RCT and its follow-up article^12,13^. In addition to DMFS, demographic background (gender, age) and oral health related behaviours (frequency of tooth brushing, use of fluoride toothpaste, snacking habits, location of treated caries lesion, presence of plaque on lesion) were included in the statistical analysis^12,13^. Inter-examiner variability was measured and kappa statistics were completed in all RCTs for examiner discrepancies.

### Study findings and outcomes

In Duangthip *et al*.^13^, three treatment groups were established to compare dentine caries arrest between SDF and NaF by employing varying application intervals (annual and 3 weekly applications) and examination time points at 6 months (m), 12 m, 18 m. Caries arrest rate was significantly higher in the SDF groups (annual and 3 weekly applications) compared to NaF (3 weekly applications) (p < 0.001, CI 95%). Duangthip *et al*.^12^ extended the latter study by adding examination time points at 24 m and 30 m. At 30 m, Duangthip *et al*.^12^ reported the caries arrest rate of Group 1 (annual SDF application) to be significantly higher than both Group 2 (3 weekly SDF application) and Group 3 (3 weekly NaF application) (p < 0.001).

Lo *et al*.^14^ found a statistically significant difference in caries arrest rate by SDF over that of NaF or water (p < 0.001). Chu *et al*.^15^ furthered the study to 30 m and found SDF groups arrested caries better than all other groups (p < 0.001) and with more arrested lesions appearing black than other groups (p < 0.001), but parental satisfaction with their child’s dental appearance and dental health did not significantly change (p < 0.05). Chu *et al*.^15^ found that soft caries removal.

Prior to SDF application did not induce a significant difference in the caries arrest rate when compared to SDF application without excavation (C.I. 95%).

Wong *et al*.^17^ and Wong *et al*.^16^ conducted secondary statistical analyses (Bayesian and multivariant model, respectively) on previously collected data in RCTs by Chu *et al*.^15^ and Lo *et al*.^14^. Wong *et al*.^16^ found that SDF resulted in a shorter arrest time than NaF. Wong *et al*.^17^ applied the Bayesian model and found that soft caries excavation resulted in a shorter caries arrest time than no excavation, and that SDF induced caries arrest time was reduced compared to NaF.

The details of all the included studies are shown in Table 2.

**Table 2:** Data extraction sheet and characteristics of included studies.

	Article	Population	Intervention	Comparison	Outcome	Drop out	Type of Study	Duration	Location	Statistical Analysis Used	Results & Findings
RCT 1	Chu *et al*.^15^	375 Children Boys 209 (56%), Girls 166 (44%) Mean age 4.0±0.8 y Upper Anterior Teeth	• Caries excavation with 38% SDF every 12 m (Gp 1) • No caries excavation with 38% SDF every 12 m (Gp 2)	• Caries excavation, and then 5% NaF applied at Day 0 and every 3 m (Gp 3) • No caries excavation, and then 5% NaF applied at Day 0 and every 3 m (Gp 4) • Water application (Gp 5)	DMFS, Caries arrest	308 remaining • Drop out rate (18%) • Gp1(20%), Gp2(19%), Gp3(18%), Gp4(16%), Gp5(15%), (Chi^2^, p > 0.05)	RCT	30 m	Guangzhou, Southern China	• Kappa • ANOVA • Scheffe’s multiple comparison (0.05) • Chi_2_ (p < 0.001) • ANCOVA • McNemar Test	• DMFS score of upper anterior teeth between baseline vs 30 m exam, not significant (p > 0.05) • intra-examiner reproducibility (Kappa >0.95) at baseline and follow up exams • No significant difference (ANOVA, p > 0.05) - Mean age, dmfs scores, number of decayed tooth surfaces, number of non-vital teeth, # new caries, # of arrested caries, increment of non-vital teeth @ each follow up exam • @24 m follow up, no significant difference between Gp1-Gp5 (Chi^2^, p > 0.05) – toothbrushing behavior, use of fluoridated toothpaste • @24 m follow up, significant difference from baseline (McNemar Test, p < 0.001) – toothbrushing behavior, use of fluoridated toothpaste • @30 m (p< 0.001) – significant different in mean # arrested carious tooth surfaces in Gp1-Gp5 • Gp1 and Gp2 had more arrested caries than all other groups (ANOVA, p < 0.001) • Gp1 and Gp2 had more arrested caries appear black than all other Gps (Chi^2^, p < 0.001) • Children in Gp5 developed more new caries lesions than all other Gps (ANOVA, p <0.001) • No significant difference in increment of non-vital teeth in all Gps • No adverse side-effects (discoloration/damage to gingival tissues) • No significant difference in parental satisfaction with their child’s dental appearance and dental health (McNemar, p <0.05) − 2/5 s of children received other dental care during study (majority performed on primary molars, unrelated to study) • Significantly more arrested caries @ 30 m Higher baseline caries (ANCOVA, p < 0.001), SDF applications (ANCOVA, p < 0.001), brushed teeth more often (ANCOVA, p = 0.003) • No difference in mean number arrested caries with excavation between • Gp1 and Gp2 (95% CI = 0.75 to −1.42) • Gp3 and Gp4 (95% CI = 0.04 to −2.12)
	Lo *et al*.^14^	375 Children Boys 209 (56%), Girls 166 (44%) Mean age 4.0±0.8 y Upper Anterior Teeth	Same as above	Same as above	DMFS, Caries arrest	• 341 remaining • Drop out rate (9.1%) similar in 5 Gps, (Chi^2^, p > 0.05) • # Attended exam/Drop out%: 6 m = 365(3%), 12 m = 353(6%), 18 m = 341(9%)	RCT	18 m	Guangzhou, Southern China	• Kappa • ANOVA • Scheffe’s multiple comparison (0.05) • Chi^2^ (p < 0.001)	• intra-examiner reproducibility: Baseline (Kappa, 0.98), 6 m (Kappa, 0.98), 12 m (Kappa, 0.95), 18 m (Kappa, 0.96) • No significant difference in all groups at baseline: DMFS, number of tooth surfaces with active caries, mean number of non-vital upper anterior teeth, mean number of retained roots (ANOVA, not significant) • No significant difference between Gp1-Gp5 (Chi^2^, p > 0.05) – toothbrushing behavior, use of fluoridated toothpaste • At all follow up exams (6 m, 12 m, 18 m)- significant difference in mean number of arrested caries among 5 Gps • Caries arrest is statistically significantly higher in SDF Groups (Gp1 and Gp2) (ANOVA, Scheffe’s multiple comparisons, p < 0.001) • Annual SDF application (Gp 1 and Gp2) higher incidence of arrested caries appearing black (P <0.001) than Gp 4, followed by Gp 3 (ANOVA, p <0.001) • No statistical difference in increment of non-vital teeth among 5 Gps • No adverse side effects observed • No difference in mean number arrested caries tooth surfaces @18 m with or without caries excavation (No significance) • Caries excavation before NaF application reduced proportion of arrested caries lesions that had become black • Caries excavation before SDF application, no significant benefit
	Wong *et al*.^17^	375 Children Boys 209 (56%), Girls 166 (44%) Mean age 4.0±0.8 y Upper Anterior Teeth	Same as above	Same as above	DMFS, Caries arrest	• Chu *et al*.^15^ 308 remaining Drop out rate (18%) • Lo *et al*.^14^ 341 remaining Drop out rate (9.1%)	Secondary analysis of RCT (Lo *et al*.^14^ and Chu *et al*.^15^)	18 m 30 m	Guangzhou, Southern China	• Bayesian model	• Both excavation groups have a shorter arrest time than non-excavation groups • SDF has shorter arrest times than NaF (w or w/o excavation)
	Wong *et al*.^16^	375 Children Boys 209 (56%), Girls 166 (44%) Mean age 4.0±0.8 y Upper Anterior Teeth	Same as above	Same as above	DMFS, Caries arrest	• Chu *et al*.^12^ 308 remaining Drop out rate (18%) • Lo *et al*.^14^ 341 remaining Drop out rate (9.1%)	Secondary analysis of RCT (Lo *et al*.,^14^ and Chu *et al*.^15^)	18 m 30 m	Guangzhou, Southern China	Multilevel modeling of correlated grouped survival data, using grouped proportional hazards models with constant regression coefficients	• Clustering effect among the arrest times from the same child was very strong (95% CI = 1.822 to 3.066) • Schools, gender and location had no significant impact on caries arrest. • SDF had shorter arrest time than NaF
RCT 2	Duangt hip *et al*.^13^	304 Children Boys 183 (60%), Girls 121 (40%) Mean age 41 ± 4 m Anterior and Posterior Teeth	30% SDF applied at 0 and 12 months (Gp 1) 30% SDF applied at 0, 7, 14 days (Gp 2)	• 5% NaF 3x applied at 0, 7, 14 days (Gp 3)	DMFS, Caries arrest	• 275 remaining • Drop out rate (9.5%) • Gp1(11%), Gp2(8%, Gp3(9%) (Chi^2^, p > 0.05)	RCT	18 m	Hong Kong (16 Kindergarten schools)	• Kappa • ANOVA • Bayesian models • Multi-level survival analysis (WinBUGS)	Exams performed at: 0, 6, 12, 18 m • No difference among Gp1, Gp2 or Gp3 in demographic background, oral health behaviors, oral hygiene status, caries experience @baseline • 18 m - Caries arrest rate, (Chi^2^, p < 0.001) ○ Gp1(40%), Gp 2(35%), and Gp3 (27%) • 6 m and 12 m - Caries arrest rate Gp2 > Gp1 • Factors significantly affecting time to caries arrest (95% C.I.) ○ Tx Gp, presence of plaque on lesions, tooth type, tooth surface • Factors NOT significantly affecting time to caries arrest (95% C.I.) ○ Demographic background, oral health related behaviors, baseline caries experience • SDF caries arrest (Gp1 or Gp2) was better than NaF at 6 m (Chi2, p < 0.001), 12 m (Chi2, p <0.001), and 18 m (Chi2, p <0.001)

### Quality assessment

All RCTs developed PICO formulated question(s) involving caries arrest by the use of SDF intervention compared to NaF. Sample size calculations were performed in Duangthip *et al*.^13^ (and also reported in the follow-up article Duangthip *et al*.^12^), but were not performed in Lo *et al*.^14^ or any of its follow-up articles. Patients were allocated into groups by stratified block randomization in Duangthip *et al*.^13^ and Duangthip *et al*.^12^, while Chu *et al*.^15^ and Lo *et al*.^14^ utilized sequential allocation. All RCTs utilised blinding protocols. While the study by Duangthip *et al*.^13^ and its follow- up article (Duangthip *et al*.^12^) adopted a triple blind protocol where the treatment providers, sole examiner and participants were blind to the intervention/control, the study by Lo *et al*.^14^ and its follow-up article (Chu *et al*.^15^) employed a double blind protocol where an independent examiner was recruited in the study. However, due to the difference in physical appearance between intervention and comparison reagents, patients, providers and/or examiners were likely able to distinguish between the intervention and comparison reagents. Otherwise, all groups were treated equally during the treatment time including the number of exams and/or treatment visits each group attended.

**Figure 2:**
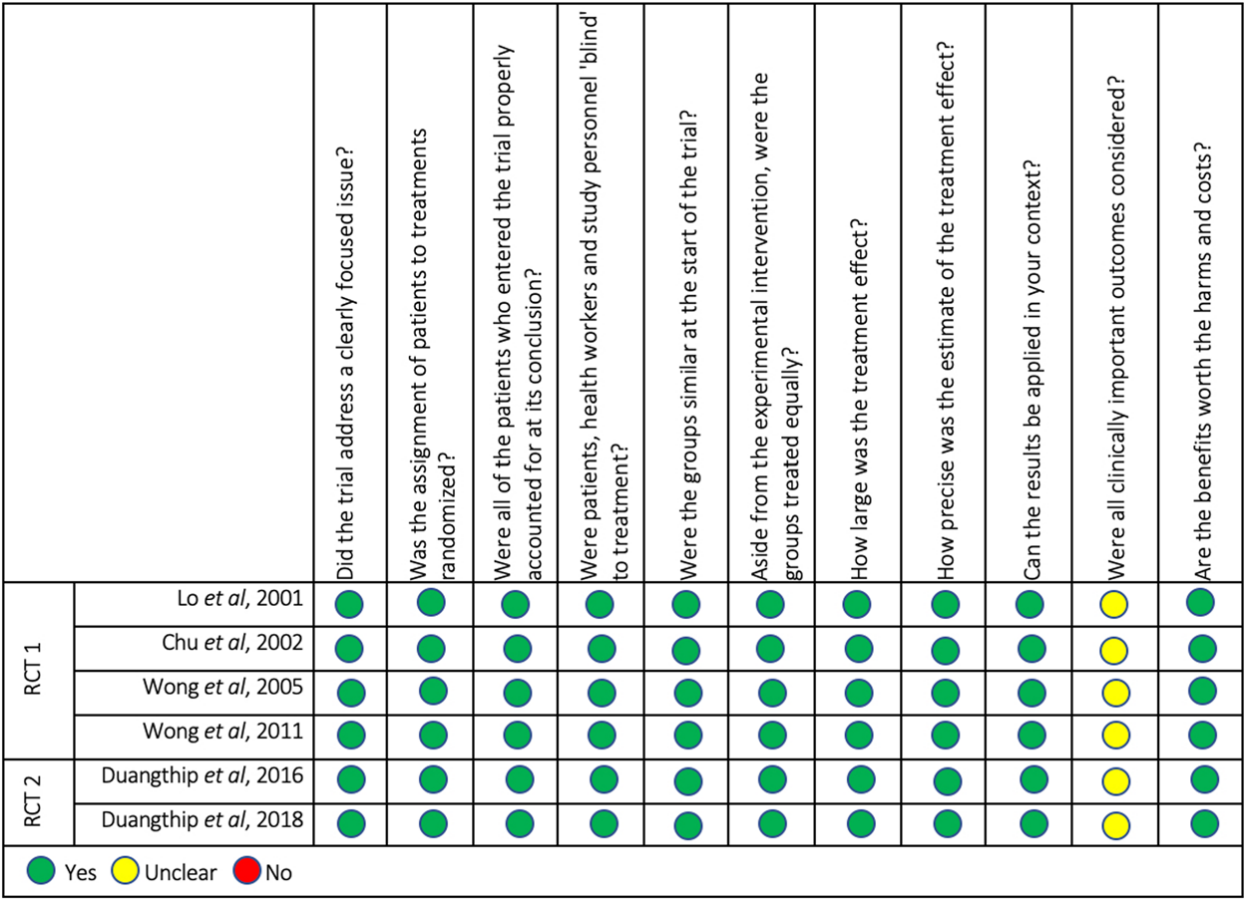
Quality assessment of the included studies by CASP tool. Summary review of the qualitative assessment of the included studies by using CASP tools for randomised controlled trials (RCTs) consisting of 11 quality criteria. Green-coded circle indicates that the study satisfactorily met the respective quality criterion, yellow-coded circle indicates that the study partially met the respective quality criterion, and the red-coded circle indicates that the study did not meet the respective quality criterion.

Clinically important outcomes were recorded and statistical analyses were conducted. Dropouts were reported in all articles. Lo *et al*.^14^ and its follow-up article (Chu *et al*.^15^) reported no statistically significant differences in the number of non-vital teeth among the five treatment groups, while Duangthip *et al*.^13^ and its follow-up article (Duangthip *et al*.^12^) reported drop out by true failure to follow up or had moved, number of teeth needing restorative treatment, and exfoliation. Duangthip *et al*.^12^ found no significant difference between all groups regarding prevalence of non-vital teeth in the International Caries Detection and Assessment System (ICDAS) 3–4 (p > 0.05). Benefits, harms, and costs regarding treatment agents were discussed in all RCTs. The overall inter-examiner variability was 92.4% for the six included studies. High concordance was observed, signifying high reproducibility between the two examiners. The details of all the included studies are shown in Fig. 2.

### Statistical meta-analysis

Two meta-analyses were conducted, encompassing: Lo *et al*.^14^, Chu *et al*.^15^, Duangthip *et al*.^13^ and Duangthip *et al*.^12^. In meta-analysis 1, arrested caries by treatment group were analyzed in the 18 m trials of Lo^14^ and Duangthip *et al*.^13^, while meta-analysis 2 compared the 30 m results^12,15^.

**Figure 3:**
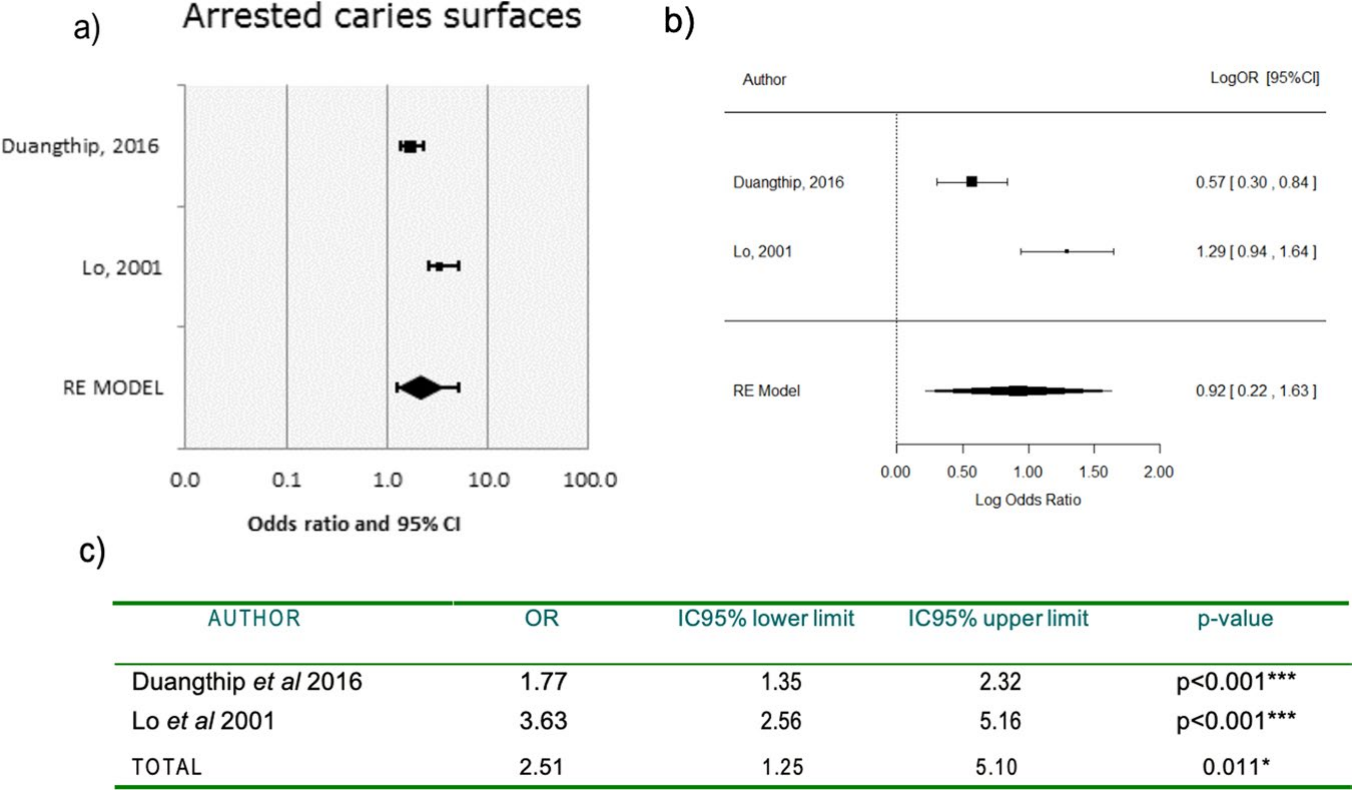
Forest and logarithmic plots for pooled ORs of arrested carious surfaces (meta-analysis 1, 18 m). Forest plot for pooled ORs of arrested caries surfaces at 18 m. (**a**) Duangthip *et al*.^13^ reported OR = 1.77, interpreting that SDF increased the probability of arresting active caries on surfaces at 77% compared to NaF, which is higher (p < 0.001). Lo *et al*.^14^ reported OR = 3.63 >1, favoring SDF (p < 0.001). Taken into consideration that OR = 1 is the reference level whereby no effect is assigned to the test treatment SDF. OR >1 means an increment of arrested caries rate in the SDF group compared to control NaF. OR < 1 means a reduction of arrested caries rate in the SDF group compared to control NaF. Logarithmic measure for pooled ORs of arrested caries surfaces at 18 m. (**b**) Taking into consideration logarithmic focus provides symmetrical confidence intervals. Log(OR) = 0 is the reference level: no effect is assigned to the SDF treatment. Log (OR) >0 means an increment of arrested caries rate in the SDF group compared to control NaF. Log (OR) <0 means a reduction of arrested caries rate in the SDF group compared to control NaF.

**Figure 4:**
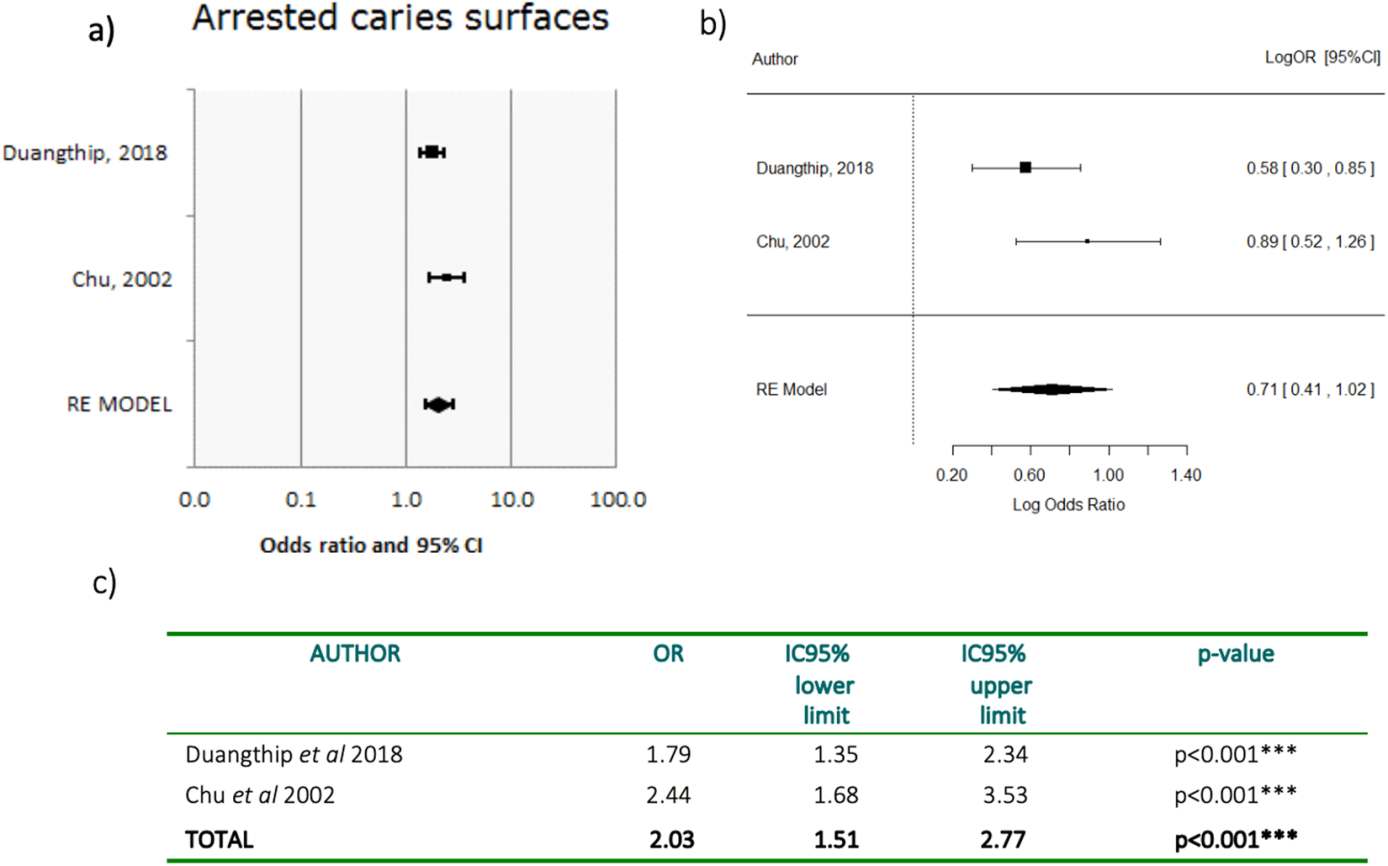
Forest and logarithmic plots for pooled ORs of arrested carious surfaces (meta-analysis 2, 30 m). Forest plot for pooled ORs of arrested caries surfaces at 30 m. (**a**) Duangthip *et al*.^13^ reported OR = 1.79, interpreting that SDF increased the probability of arresting active caries on surfaces at 79% compared to NaF, which is higher (p < 0.001). Chu *et al*.^15^ reported OR = 2.44 > 1, favoring SDF (p < 0.001). Taken into consideration that OR = 1 is the reference level whereby no effect is assigned to the test treatment SDF. OR >1 means an increment of arrested caries rate in the SDF group compared to control NaF. OR <1 means a reduction of arrested caries rate in the SDF group compared to control NaF. Logarithmic measure for pooled ORs of arrested caries surfaces 30 m. Taken into consideration logarithmic focus provides symmetrical confidence intervals. Log(OR) = 0 is the reference level: no effect is assigned to the SDF treatment. Log (OR) >0 means an increment of arrested caries rate in the SDF group compared to control NaF. Log (OR) <0 means a reduction of arrested caries rate in the SDF group compared to control NaF.

Data between studies was harmonized to determine the odd ratio (OR). Individual estimated ORs and the pooled ORs were generated and revealed a significant increased probability of SDF arresting caries as compared to NaF at both 18 m (OR = 2.51, 95% CI:1.2,5.10, p = 0.011) and 30 m (OR = 2.03, 95% CI: 1.5,2.77, p < 0.001). Please see Figs 3 and 4.

#### Heterogeneity analysis

**Figure 5:**
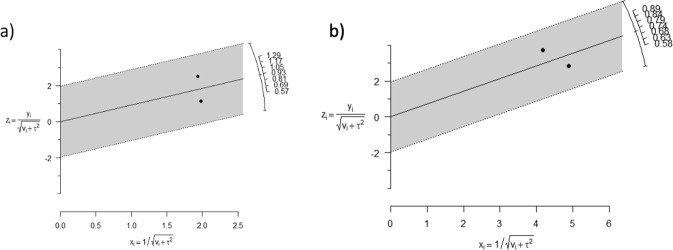
Galbraith graphs for analysis of heterogeneity. Galbraith graphs for heterogeneity analysis at (**a**) 18 m (I2 = 90.3, Q = 10.29, p = 0.001), both studies are distanced but between the confidence levels and (**b**) 30 m (I2 = 44.3%, Q = 1.79; p = 0.180), both studies are distanced but between the confidence levels. Taken into consideration the horizontal axis corresponds to precision of the study and the vertical axis to standardized effect. The arch shows ln(OR) and the central line is the global effect measure.

Heterogeneity was evaluated with Cochran’s Q Test and the I^2^ Index. A higher level of heterogeneity was found at 18 m (I^2^ = 90.3, Q = 10.29, p = 0.001) than at 30 m (I^2^ = 44.3%, Q = 1.79; p = 0.180). Galbraith graphs (See Fig. 5) were also generated to establish heterogeneity of the meta-analyses. Both meta-analysis 1 and meta-analysis 2 fell within confidence levels suggesting distance but lay within the confidence intervals.

#### Potential publication bias

**Figure 6:**
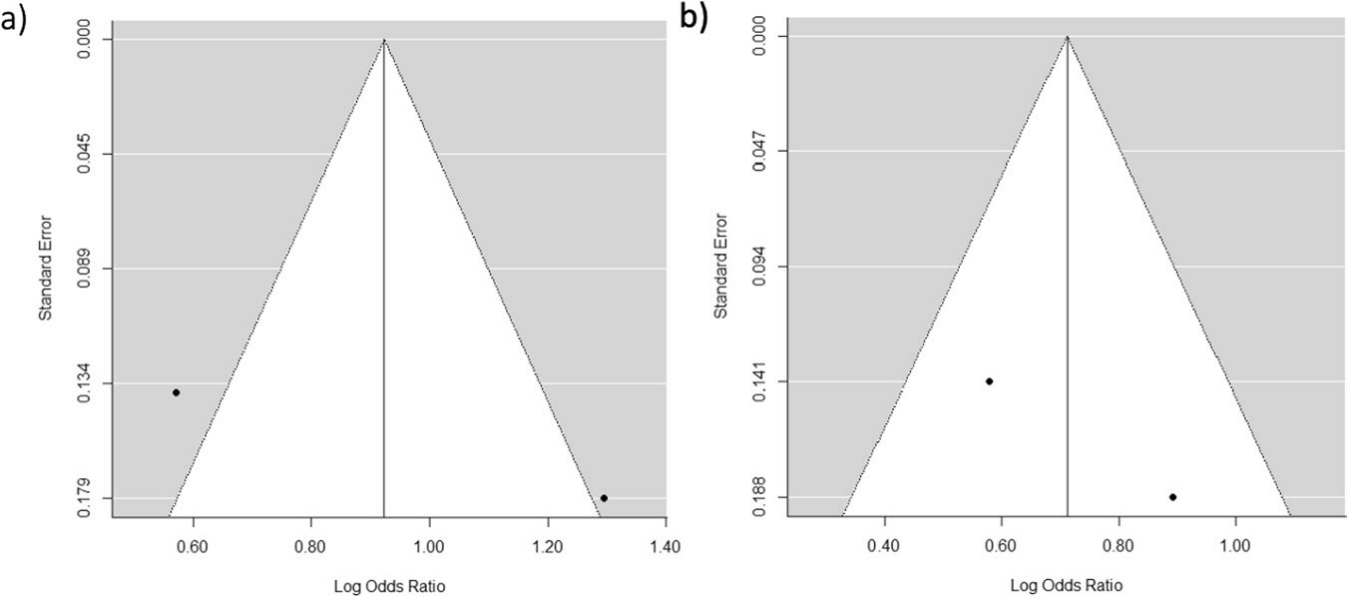
Funnel plots for publication bias. Funnel plots for publication bias at (**a**)18 m Duangthip *et al*.^13^ is vertically higher, exhibiting a higher precision, due to its larger sample size with acceptable symmetry for this precision level and (**b**) 30 m Duangthip *et al*.^12^ is vertically higher, exhibiting a higher precision due to larger sample size. Taken into consideration that an acceptable symmetry at this precision level, there are some articles reporting different effects regarding effectiveness of SDF to NaF.

Publication bias was evaluated with funnel graphs (See Fig. 6). Duangthip *et al*.^13^ and Duangthip *et al*.^12^ reported a larger sample size and thus increased precision, but both meta-analysis 1 and meta-analysis 2 showed acceptable symmetry. No publication bias was detected.

## Discussion

Dental caries is one of the most common chronic childhood diseases^2^. It is therefore essential to identify approaches for the management of dental caries that are minimally invasive and less traumatic, such as water fluoridation, fluoridated toothpastes, topical fluoride varnishes^18^. Fluoride interferes with microbial processes of the oral biofilm and inhibits demineralization^19^. Thus fluoride is not only a preventive mean to reduce caries prevalence, but can also partake in caries arrest. The results of this meta-analyses found that SDF, when compared to NaF, was a more effective fluoride containing reagent for dentine caries arrest in children.

After reviewing the published literature, six articles met inclusion criteria for this systematic review. However, only four articles were considered for meta-analyses comprising two individual RCTs, both of which reported results at 18 m^13,14^ and at 30 m^12,15^ time points. Articles excluded from the meta-analysis were due to 1) different reported effect measures and 2) different group comparisons. Duangthip *et al*.^13^ provided hazard ratios, whereas Wong *et al*.^17^ estimated relative risk, both studies could not be analyzed due to the limited number of studies and lack of raw data. Also, Duangthip *et al*.^13^ directly compared SDF to NaF, whereas Wong *et al*. 2011 compared intervention groups (SDF or NaF) to controls and estimated the time-varying regression coefficients that Duangthip *et al*.^13^ did not mention.

The quality of each study was critically appraised using CASP protocols for RCTs. Sample size calculations were not reported by Lo *et al*.^14^ and its follow-up article (Chu *et al*.^15^) so the quality of their papers is therefore compromised as the sample may not have been a true representation of the population. In general, the articles met most CASP criteria with the aforementioned exceptions, meaning their level of evidence was high and thus supporting their outcomes.

The findings of this systematic review need to be viewed with caution as the study encountered limiting factors. For instance, all RCTs were conducted in Guangzhou, China and Hong Kong, China, which represent a very specific demographic with a diet, hygiene regimen, and water fluoridation different from the western culture. All RCTs reported blind randomization. While the study by Duangthip *et al*.^13^ and its follow-up article (Duangthip *et al*.^12^) adopted a triple blind protocol where the treatment providers, sole examiner and participants were blind to the intervention/control, the study by Lo *et al*.^14^ and its follow-up article (Chu *et al*.^15^) employed a double blind protocol where an independent examiner was recruited in the study. However, SDF and NaF differ in both colour and texture and could have been discerned by examiner and treatment provider, therefore compromising their^12–15^ ability to treat all group equally. This could play a role in reporting the outcomes of the studies^12–17^. Within the meta-analyses, only those in English were included, which could limit potential foreign publications. Although six articles were identified, all included studies are based on two clinical trials. It would be ideal to conduct further clinical trials to formulate stronger clinical recommendations.

In summary, SDF is more effective as a dentine caries arresting reagent than NaF and has many implications for pediatric dentistry. There is a reduced treatment cost associated with SDF/Fluoride varnish use^20^. The ease of application can result in greater delivery of the reagent to a larger population of children with untreated caries. The main reported disadvantage being the non-aesthetic black colouring of carious lesions after SDF application^12–15^, however the additional use of potassium iodide has been reported to reduce the discolouration^21^. Surveys report a higher parental acceptance of SDF associated black staining on posterior teeth than anterior teeth, additional factors such as behavioral barriers, socioeconomic status, and indications for sedation and/or hospital dentistry also increased parental acceptance^22^. Though the quality of evidence and meta-analyses are strong, the findings were only based on 2 studies. Further studies are needed to evaluate the minimal necessary concentration and frequency of application to arrest dentine caries of primary and permanent teeth.

## Conclusion

In view of the findings in this systematic review, SDF is a more effective dentine caries arresting agent than NaF. However, these findings need to be viewed with caution as further clinical research is needed to consolidate findings.

## Methods

The Preferred Reporting Items for Systematic reviews and Meta-Analysis (PRISMA) guidelines were adopted for the current study.

### Search strategy and eligibility criteria

Studies that investigated the caries arrest potential of SDF and NaF were identified by using a search strategy in the following electronic databases: PubMed, Web of Science, Ovid, and Cochrane Library to March 31st, 2018. Search terms were listed as follows: “silver diamine fluoride” or “silver fluoride” or “SDF” or “diamine fluoride” and “fluoride varnish” or “sodium fluoride” or “sodium varnish” and “dental caries” or “dentin caries” or “dentine caries” or “dental cavity” or “carious lesion” or “caries” and “caries arrest” or “dental caries activity test” or “DMF” or “decayed missing filled” and “child” or “children” or “pediatric”. The reference lists from the retrieved articles were reviewed, and hand-searched the following journals: Caries Research, Journal of Dentistry, Journal of Dental Research, Journal of Oral Health and Preventive Dentistry, and Pediatric Dentistry (to March 31st 2018) for further relevant studies.

The search strategy yielded 107 articles by using electronic and hand searching. However, only six articles were then subjected to full-text qualitative and quantitative analyses as described in the results section. Please refer to Fig. 1 for the selection process and Table 1 for inclusion and exclusion criteria.

### Data extraction and quality assessment

A preset data sheet was developed to extract information from the included studies. From each included study, the following data were extracted: article, population, intervention, comparison, outcome, drop out, type of study, duration, location, statistical analysis, results and findings. Please refer to Table 2. All data was extracted by two reviewers (Trieu and Mohamed). However, disagreements between the two investigators were resolved by a third independent reviewer (Lynch). The quality of the included studies was critically appraised by the CASP for randomized clinical trials (RCT). This protocol consists of a set of eleven questions addressing key aspects of the quality of RCTs, including: question formulation, type of study, relevancy, quality, results, precision of results, application of results to populations, outcomes, and the evaluation between benefits over harms and costs of studies. For detailed information, please see Fig. 2. Inter- examiner variability among the 2 examiners was measured by calculating the percentage of agreement (%) among the 2 examiners in each CASP checklist criteria.

### Statistical analysis

Pooled measurements were calculated (OR with 95% CI) by random effects model (REM) to assess the strength of association between SDF and NaF to arrest caries. A study of heterogeneity was performed in order to assess the variability between included studies using Cochran’s Q test and I^2^ Index. Consistency of results from different authors were explored with Galbraith’s graphs. The Funnel graphs have also been performed to assess potential publication bias. The software R 3.0.2 and its ‘metafor’ package was used to perform the meta-analyses. The level of significance used in the analysis has been 5% (α = 0.05).
